# Undersized lung grafts in chronic obstructive pulmonary disease transplant recipients: risk factor or acceptable compromise?

**DOI:** 10.3389/ti.2026.16246

**Published:** 2026-07-01

**Authors:** Yujiro Kubo, Juan José Giron, Guillermo Rodríguez Dávila, Felipe Alayza Avendaño, Diletta Mongiello, Pablo Cordero Iglesias, Alejandra Romero Román, Silvana Crowley, Andrea Mariscal, José Manuel Naranjo Gómez, Nuria María Novoa Valentín, David Gomez-de-Antonio

**Affiliations:** 1 Thoracic Surgery Department Hospital Universitario Puerta de Hierro Majadahonda, Madrid, Spain; 2 Surgery Department, School of Medicine, Universidad Autónoma de Madrid, IDIPHISA, Madrid, Spain

**Keywords:** chronic obstructive lung disease, overall survival, predicted total lung capacity, primary graft dysfunction, size matching

## Abstract

Size matching is an important concern during lung allocation for lung transplantation (LTx). Although some mismatch is accepted—often favoring larger grafts for patients with chronic obstructive pulmonary disease (COPD)—the optimal strategy remains unclear. This study evaluated the impact of graft size mismatch on postoperative outcomes in COPD. We retrospectively reviewed 130 of 491 patients who underwent LTx at a single center between January 2013 and December 2023. Patients were classified into three groups based on donor-to-recipient predicted total lung capacity ratio: undersized (<0.9), size-matched (0.9–1.1), and oversized (≥1.1). Donor and recipient characteristics were collected. Primary graft dysfunction (PGD) was the primary endpoint; overall survival was secondary. Of the 130 patients, 17 (13%) received undersized grafts, 67 (52%) size-matched grafts, and 46 (35%) oversized grafts. The undersized group had a lower incidence of PGD at 48 and 72 h than other groups (*p* < 0.01 and *p* = 0.02). Grade 3 PGD at 72 h was less frequent among groups (p = 0.03). Overall survival was higher in the undersized group (*p* = 0.018 and *p* = 0.045). Oversized grafts may not be optimal for LTx in COPD. Undersized grafts appear to be a viable strategy.

## Graphical Abstract

**Figure:**
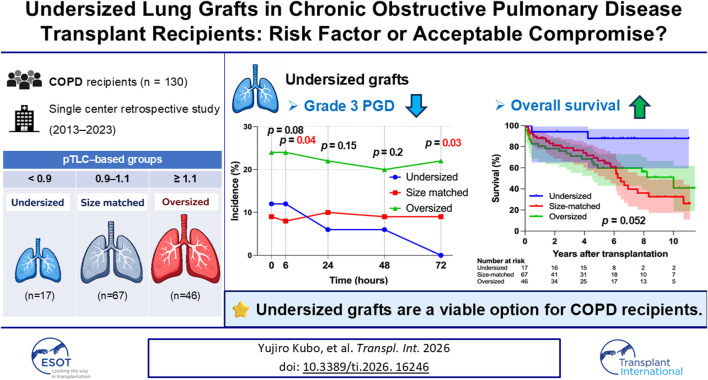
Graphical abstract of the study. In 130 COPD recipients undergoing bilateral lung transplantation, recipients were divided into the following pTLC-based groups: undersized (<0.9, n = 17), size-matched (0.9–1.1, n = 67), and oversized (≥1.1, n = 46). The undersized group showed lower incidences of grade 3 PGD compared with the other groups. Overall survival was significantly higher in the undersized group compared with the size-matched and oversized groups (*p* = 0.018 and *p* = 0.045, respectively). COPD, chronic obstructive pulmonary disease; PGD, primary graft dysfunction; pTLC, predicted total lung capacity.

## Introduction

Chronic obstructive pulmonary disease (COPD) is a progressive and irreversible respiratory disorder characterized by persistent airflow limitation, often culminating in respiratory failure. Lung transplantation (LTx) serves as a definitive therapy for patients with end-stage COPD. Among the multiple factors influencing post-transplant outcomes, donor-recipient lung size matching has been recognized as an important consideration.

While size-matching strategies have traditionally aimed to minimize graft-recipient mismatch, the clinical impact of oversizing or undersizing remains a matter of ongoing debate. Some studies suggest that oversized lungs may offer functional advantages in COPD patients [1–3], whereas excessive oversizing may increase the risk of complications such as delayed chest closure, impaired ventilation, and postoperative infections [1, 4]. Nonetheless, the evidence remains inconclusive, and real-world data regarding the prognostic significance of size mismatch—particularly in the context of COPD—are still limited.

This study aimed to evaluate the impact of donor lung size mismatch on perioperative and long-term outcomes in patients with COPD who underwent bilateral LTx at a single center.

## Materials and methods

### Patient selection

**FIGURE 1:**
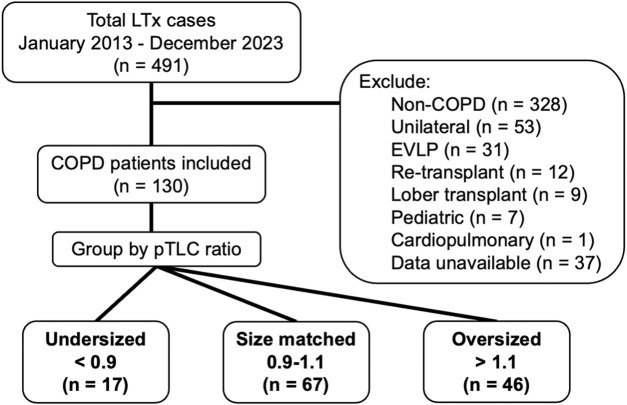
Flowchart of patient selection. Among 491 lung transplants performed between January 2013 and December 2023, 130 patients with chronic obstructive pulmonary disease (COPD) who received bilateral lung transplantation were included. Exclusions comprised non-COPD cases, unilateral or lobar transplants, re-transplants, pediatric or cardiopulmonary transplants, cases with incomplete data, and *ex vivo* lung perfusion (EVLP). Groups were classified by predicted total lung capacity (pTLC) ratio: undersized (<0.9), size-matched (0.9–1.1), and oversized (≥1.1).

This retrospective study was conducted at [Author information withheld for anonymized peer review]. We reviewed data from 491 lung transplants performed between January 2013 and December 2023 (Figure 1). After exclusion of non-COPD cases, unilateral transplants, *ex vivo* lung perfusion (EVLP), re-transplantation, lobar transplants, pediatric recipients, cardiopulmonary transplants, and cases with incomplete data, a total of 130 COPD patients were included in the analysis. Patients were divided by predicted total lung capacity (pTLC) donor-to-recipient ratio into undersized (<0.9), size-matched (0.9–1.1), and oversized (≥1.1) groups, including 17 undersized, 67 size-matched, and 46 oversized patients. pTLC was calculated using equations established by the European Respiratory Society in 1995 [5]. For men, pTLC (L) = (7.99 × height [m] − 7.08); for women, pTLC (L) = (6.60 × height [m] − 5.79). The pTLC ratio thresholds were selected based on previous reports and our institutional experience, reflecting clinically relevant differences in outcomes across these ranges [6–8]. This retrospective study was approved by the Institutional Review Board of [Author information withheld for anonymized peer review] (approval number: PI 159/25, 7 July 2025). The requirement for informed consent was waived due to the retrospective nature of the study.

### Organ allocation policy and recipient selection

In Spain, the allocation of lungs is coordinated at the national level by the National Transplant Organization (ONT). Donated lungs are offered to candidate hospitals in order, considering the distance from the donor location. In selecting recipients, our institute considers criteria such as urgency, blood group compatibility, clinical factors (including age, infection status and hemodynamics), and size matching between donor and recipient. Size mismatch was not intentionally selected based on recipient characteristics, reflecting real-world allocation practices.

### Surgical technique, perioperative management, and postoperative follow-up

Bilateral lung transplantation was performed utilizing the sequential technique. Each native lung was removed one at a time via the clamshell or bilateral thoracotomy, and donor lungs were transplanted sequentially. Anastomoses of the bronchi, pulmonary arteries, and left atrium were performed with conventional methods. Extracorporeal life support (ECLS) was instituted when clinically indicated, including in cases of severe pulmonary hypertension, hemodynamic instability, hypoxemia, or hypercapnia. For oversized grafts, chest closure was performed cautiously to avoid graft compression, with consideration of delayed chest closure or graft size reduction when necessary. Postoperative ventilatory management was based on a lung-protective strategy, including low tidal volume ventilation and appropriate positive end-expiratory pressure, with early extubation whenever feasible. The immunosuppressive therapy includes basiliximab (20 mg) on Days 1 and 4 post-operatively, and a life-time triple immunosuppression regimen based on tacrolimus, mycophenolate mofetil and prednisone. Prophylactic antibiotic therapy is adjusted based on the bacterial species isolated from specimens taken from the donor and recipient at the time of surgery. Empirical administration of broad-spectrum antibiotics should be terminated after 7–10 days. For prophylactic antifungal therapy, liposomal amphotericin B (6 mL) is administered via nebulizer every 48 h until discharge, and once weekly thereafter. Follow-up included regular clinical visits, laboratory testing with monitoring of immunosuppressant levels, and spirometry. Visit frequency decreased progressively over time, from weekly after discharge to quarterly in the long term.

### Study design

Demographic data collected for recipients included age, sex, height, body mass index (BMI), lung allocation score (LAS), secondary pulmonary hypertension, smoking history, cytomegalovirus (CMV) infection, previous thoracic surgery, arterial hypertension, diabetes mellitus, dyslipidemia, gastroesophageal, reflux disease, coronary artery disease, anti-human leukocyte antigen (HLA) antibodies, forced expiratory volume in 1 s (FEV_1_), pretransplant medical condition—such as extracorporeal membrane oxygenation (ECMO), invasive mechanical ventilation, or non-invasive mechanical ventilation—intensive care unit stay prior to LTx, and pTLC.

For each recipient, donor demographic characteristics were also collected: age, sex, sex mismatch, height, BMI, type of donor, cause of death, duration of mechanical ventilation, arterial partial pressure of oxygen over fraction of inspired oxygen (PaO_2_/FiO_2_), smoking history, and pTLC.

Perioperative and postoperative outcomes included ischemic times for each lung, use of extracorporeal life support (ECLS), primary graft dysfunction (PGD) grading at 0, 6, 24, 48, and 72 h post-transplantation, rethoracotomy, postoperative ECMO, tracheostomy, duration of mechanical ventilation, intensive care unit (ICU) and hospital stay, and in-hospital mortality. Long-term outcomes included development of chronic lung allograft dysfunction (CLAD), time to CLAD onset, and survival at 30 days, 1 year, 3 years, and 5 years. PGD was assessed according to the International Society for Heart and Lung Transplantation (ISHLT) criteria (2005 consensus definition, revised 2017) [9, 10].

The incidence of PGD was analyzed as the primary outcome, and overall survival as the secondary outcome.

### Statistical analysis

All statistical analyses were performed using GraphPad Prism 10 Software version 10.4.2 (GraphPad Software, Inc., San Diego, CA) and EZR version 1.68 (Saitama Medical Center, Jichi Medical University, Saitama, Japan), which is a graphical user interface for R version 4.4.2 (The R Foundation for Statistical Computing, Vienna, Austria) [11]. Statistical significance was evaluated using the Student’s t-test or the Mann–Whitney U-test for comparisons between two groups of continuous variables, depending on the normality of data distribution; one-way ANOVA followed by Tukey’s *post hoc* test or the Kruskal–Wallis test for comparisons among multiple groups of continuous variables; and Fisher’s exact test or the chi-squared test for categorical variables, depending on the size of the groups. Overall survival was defined as the period from transplantation to death from any cause, with patients who were still alive at the last follow-up considered censored. Kaplan–Meier estimates were used to generate survival curves, and differences between groups were assessed using the log-rank test. Multivariable analysis was performed using a Cox proportional hazards regression model, including variables considered clinically relevant. Statistical significance was defined as *p* < 0.05.

## Results

### Recipient characteristics

Recipient characteristics are shown in Table 1. The median pTLC ratios were 0.84 (IQR 0.82–0.89) in the undersized group, 1.02 (IQR 0.99–1.07) in the size-matched group, and 1.27 (IQR 1.16–1.36) in the oversized group. The oversized group had a higher proportion of female recipients (only 35% male; *p* < 0.001) and was significantly shorter (median height: 158 cm) with lower pTLC (4.70 L), contributing to graft oversizing. Secondary pulmonary hypertension was most frequent in the undersized group (*p* = 0.026). Other baseline factors were comparable across groups.

**TABLE 1:** Recipient characteristics.

Variables	Undersized (n = 17)	Size matched (n = 67)	Oversized (n = 46)	*p* value
Age (years)	61 (51–69)	59 (40–69)	59 (27–70)	0.72
Sex	​	​	​	​
Male	16 (94)	46 (69)	16 (35)	**<0.001**
Female	1 (6)	21 (31)	30 (65)	​
Height (mm)	169 (158–185)	170 (156–183)	158 (147–184)	**<0.001**
BMI (kg/m^2^)	24 (18–28)	25 (17–30)	23 (18–31)	0.82
Lung allocation score	33 (30–50)	33 (28–66)	33 (26–55)	0.61
Pretransplant SPH	15 (88)	41 (61)	37 (80)	**0.026**
Smoking history	16 (94)	56 (83)	38 (83)	0.66
CMV infection	16 (94)	57 (88)	44 (96)	0.38
Prev. thoracic surgery	0 (0)	5 (8)	1 (2)	0.41
Arterial hypertension	8 (47)	19 (31)	11 (26)	0.27
Diabetes mellitus	1 (6)	6 (9)	3 (7)	0.90
Dyslipidemia	6 (35)	13 (21)	13 (31)	0.34
GERD	1 (6)	2 (3)	2 (5)	0.84
CAD	1 (6)	7 (11)	4 (10)	1
Anti-HLA antibodies	2 (12)	3 (5)	1 (2)	0.22
FEV_1_ (%)	27 (14–78)	25 (14–75)	28 (15–86)	0.66
Bridge to transplant	​	​	​	0.26
ECMO	0 (0)	0 (0)	0 (0)	​
IMV	1 (6)	1 (2)	0 (0)	​
NIMV	0 (0)	2 (3)	0 (0)	​
ICU before LTx	1 (6)	1 (2)	0 (0)	0.37
pTLC (L)	6.42 (5.36–7.70)	6.50 (4.51–7.54)	4.70 (3.91–7.22)	**<0.001**
pTLC ratio, median (IQR)	0.84 (0.82–0.89)	1.02 (0.99–1.07)	1.27 (1.16–1.36)	**<0.001**

Data are presented as median (range) or n (%).

Bold values represent statistical significance (*p* < 0.05).

BMI, body mass index; CAD: coronary artery disease; CMV, cytomegalovirus; ECMO, extracorporeal membrane oxygenation; FEV1, forced expiratory volume in 1 s; GERD, gastroesophageal reflux disease; HLA, anti-human leukocyte antigen; ICU, intensive care unit; IMV, invasive mechanical ventilation; IQR, interquartile range; LTx, lung transplantation; NIMV, non-invasive mechanical ventilation; Prev. thoracic surgery, previous thoracic surgery; SPH, secondary pulmonary hypertension; pTLC, predicted total lung capacity.

### Donor characteristics

Donor data are summarized in Table 2. Male donors were more common in the oversized group (72%; *p* < 0.001), corresponding to higher male-to-female mismatches and higher donor pTLC (median: 6.54 L). Sex mismatch patterns varied significantly (*p* ≤ 0.001). No significant differences were found in other donor variables.

**TABLE 2:** Donor characteristics.

Variables	Undersized (n = 17)	Size matched (n = 67)	Oversized (n = 46)	p value
Age (years)	57 (18–76)	54 (16–78)	57 (20–80)	0.46
Sex	​	​	​	​
Male	1 (6)	34 (51)	33 (72)	**<0.001**
Female	16 (94)	33 (49)	13 (28)	​
Sex mismatch	5 (29)	8 (12)	17 (37)	**<0.01**
Female-to-male	5 (29)	6 (9)	0 (0)	**0.001**
Male-to-female	0 (0)	2 (3)	17 (37)	**<0.001**
Height (mm)	170 (155–180)	175 (158–190)	175 (147–195)	0.13
BMI (kg/m^2^)	26 (21–33)	26 (17–37)	25 (16–34)	0.72
Type of donor	​	​	​	​
DBD	15 (88)	48 (72)	35 (76)	0.40
DCD	2 (12)	19 (28)	11 (24)	​
Cause of death	​	​	​	0.57
CVA	12 (71)	42 (63)	33 (72)	​
Trauma	4 (24)	11 (16)	5 (11)	​
Anoxia	1 (6)	7 (10)	2 (4)	​
Duration of MV (days)	2 (1–15)	2 (1–19)	2 (0–28)	0.52
PaO2/FiO2	504 (334–608)	445 (260–719)	432 (312–700)	0.40
Smoking history^a^	7 (41)	21 (34)	17 (39)	0.79
pTLC (L)	5.43 (4.44–6.09)	6.42 (4.64–8.10)	6.54 (4.67–8.50)	**0.001**
Graft size reduction	0 (0)	3 (4)	1 (2)	1

Data are presented as median (range) or n (%).

Bold values represent statistical significance (p < 0.05).

BMI, body mass index; CVA, cerebrovascular accident; DBD, donation after brain death; DCD, donation after circulatory death; MV, mechanical ventilation; PaO2/FiO2, arterial partial pressure of oxygen over fraction of inspired oxygen; pTLC, predicted total lung capacity.

^a^ Seven cases with missing data were excluded.

### Perioperative and postoperative outcomes

**FIGURE 2:**
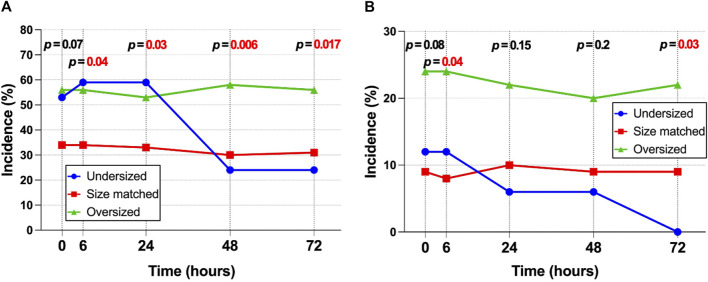
Incidence and temporal distribution of primary graft dysfunction (PGD) following lung transplantation. **(A)** Distribution of PGD grades (0–3) over time. **(B)** Incidence of Grade 3 PGD at each postoperative time point.

The incidence of PGD, the primary outcome of this study, is shown in Figure 2; Table 3. As illustrated in Figure 2A, both the undersized and oversized groups showed significantly higher PGD rates than the size-matched group at 6 and 24 h post-transplantation (*p* = 0.04 and *p* = 0.03, respectively). At later time points, only the oversized group remained significantly higher (*p* = 0.006 at 48 h; *p* = 0.017 at 72 h). Figure 2B presents grade 3 PGD incidence. Although early-phase differences were not significant (*p* = 0.08 at 0 h; *p* = 0.15 at 24 h; *p* = 0.2 at 48 h), the oversized group consistently showed higher rates. At 72 h, the undersized group had significantly lower incidence compared across the three groups (*p* = 0.03).

**TABLE 3:** Perioperative and postoperative outcomes.

Variables	Undersized (n = 17)	Size matched (n = 67)	Oversized (n = 46)	*p* value
Ischemic time				
First lung	300 (190–967)	390 (180–1035)	315 (150–819)	**0.008**
Second lung	400 (280–590)	490 (270–1120)	440 (240–933)	**0.009**
Intraoperative ECLS	7 (41)	36 (54)	22 (49)	0.42
ECMO	6 (35)	30 (45)	14 (30)	​
CPB	1 (6)	6 (9)	8 (17)	​
PGD				
0 h	9 (53)	23 (34)	25 (56)	0.07
6 h	10 (59)	23 (34)	25 (56)	**0.04**
24 h	10 (59)	22 (33)	24 (53)	**0.03**
48 h	4 (24)	20 (30)	26 (58)	**0.006**
72 h	4 (24)	21 (31)	25 (56)	**0.017**
PGD grade 3				
0 h	2 (12)	6 (9)	11 (24)	0.079
6 h	2 (12)	5 (8)	11 (24)	**0.04**
24 h	1 (6)	7 (10)	10 (22)	0.15
48 h	1 (6)	6 (9)	9 (20)	0.2
72 h	0 (0)	6 (9)	10 (22)	**0.03**
Rethoracotomy	1 (6)	8 (12)	6 (13)	0.86
Postoperative ECMO	2 (12)	6 (9)	5 (11)	0.85
VA ECMO	0 (0)	0 (0)	2 (4)	0.24
VV ECMO	2 (12)	6 (9)	3 (7)	0.68
Delayed chest closure	0 (0)	0 (0)	1 (2)	0.49
Acute rejection	9 (53)	33 (49)	25 (56)	0.91
Tracheostomy	3 (18)	16 (24)	11 (24)	0.92
Airway complication	11 (24)	22 (33)	4 (24)	0.56
Mechanical ventilation (days)	2 (1–40)	1 (1–50)	2 (1–159)	0.19
ICU stay (days)^a^	6 (4–42)	7 (2–54)	8 (2–43)	0.98
Hospital stay (days)^a^	51 (27–102)	44 (26–176)	38 (9–110)	0.69
In-hospital mortality	1 (6)	4 (6)	7 (15)	0.22
CLAD	4 (24)^b^	17 (25)	7 (15)	0.43
BOS	2 (12)	15 (22)	7 (15)	1
RAS	0 (0)	2 (3)	0 (0)	​
Time to CLAD onset (years)	3.9 (0.84–7.2)	2.5 (0.29–6.9)^c^	2.6 (0.86–7.2)	0.41
30-Day survival^d^	17 (100)	67 (100)	44 (96)	0.23
1-Year survival^d^	16 (94)	59 (88)	37 (80)	0.32
3-Year survival^d^	15 (94)	34 (72)	30 (71)	0.18
5-Year survival^d^	14 (88)	27 (60)	20 (54)	0.05

Data are presented as median (range) or n (%).

Bold values represent statistical significance (*p* < 0.05).

BOS, bronchiolitis obliterans syndrome;CPB, cardiopulmonary bypass; ECLS, extracorporeal life support; ECMO, extracorporeal membrane oxygenation; ICU, intensive care unit; PGD, primary graft dysfunction; RAS, restrictive allograft syndrome; VA, veno-arterial; VV, veno-venous.

^a^ Among patients who survived to discharge.

^b^ Two cases with missing data were excluded.

^c^ One case with missing data was excluded.

^d^ Cases with insufficient follow-up duration were excluded.

Other perioperative and postoperative outcomes are summarized in Table 3. The first and second graft ischemic times were significantly longer in the size-matched group compared to the other groups (*p* = 0.008 and *p* = 0.009, respectively). In contrast, no significant differences were observed regarding intraoperative ECLS, postoperative ECMO, tracheostomy, rethoracotomy, ventilation duration, ICU stay, or hospital stay. Mid-to long-term outcomes—including in-hospital mortality, CLAD incidence, and time to CLAD onset—were also comparable across groups.

### Survival analysis

**FIGURE 3:**
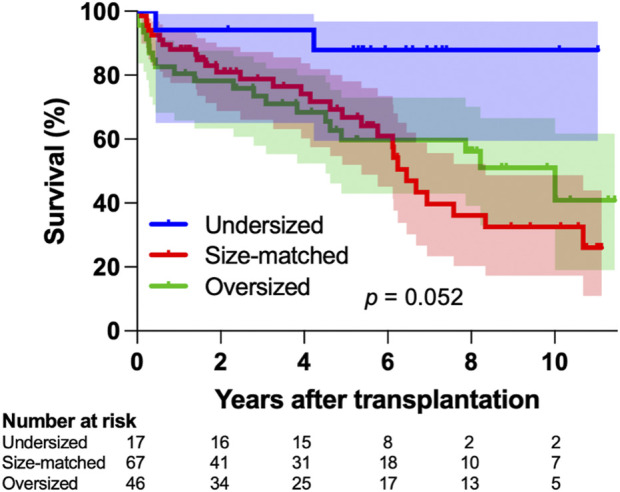
Kaplan–Meier survival curves according to donor-to-recipient lung size group. The colored area indicates the 95% confidence interval.

Overall survival curves stratified by donor-to-recipient lung size group are shown in Figure 3. The median follow-up period was 6.23 years, estimated utilizing the reverse Kaplan-Meier method. Although the three-group comparison showed no statistically significant difference (*p* = 0.052), the undersized group demonstrated a favorable survival trend. Notably, pairwise comparisons revealed significantly better survival in the undersized group compared to both the size-matched (*p* = 0.018) and oversized groups (*p* = 0.045).

Furthermore, although the sample size was small, we performed a multivariate Cox regression analysis based on the available data variables (Table 4). The significance of the undersized group was maintained in the multivariate analysis as well.

**TABLE 4:** Multivariable Cox regression analysis for overall survival.

Variable	Hazard ratio	95% CI	*p* value
Group			
Oversized	0.83	0.45–1.55	0.57
Undersized	0.14	0.03–0.64	**0.01**
Donor age	1.02	1.00–1.05	0.08
DCD donor	0.62	0.29–1.33	0.22
Urgent case	2.26	0.50–10.35	0.29
Sex mismatch	1.32	0.66–2.62	0.43
Recipient sex	1.09	0.53–2.28	0.81
Donor smoking history	1.46	0.38–5.58	0.58

Undersized grafts were independently associated with improved overall survival, whereas oversized grafts were not significantly associated with survival.

Bold values represent statistical significance (*p* < 0.05).

CI, confidence interval; DCD, donation after circulatory death.

## Discussion

This retrospective study investigated the impact of donor-to-recipient pTLC mismatch on clinical outcomes following bilateral lung transplantation for COPD. Notably, our findings revealed a significantly higher incidence of early PGD in both undersized and oversized graft groups compared to the size-matched group. Despite this, the undersized group demonstrated favorable long-term survival, with significantly better outcomes in pairwise comparisons against both the size-matched and oversized groups.

Previous study in patients with pulmonary fibrosis have reported that oversized grafts may be associated with worse early outcomes and impaired long-term survival [12]. However, the physiological implications of size mismatch likely differ between fibrotic and emphysematous lungs, due to differences in thoracic cavity compliance and native lung volume [2, 13]. Our findings suggest that in the context of COPD, modest undersizing may be considered a viable option.

The association between oversized grafts and increased PGD incidence aligns with prior studies suggesting that excessive lung size may contribute to ventilation-perfusion (V/Q) mismatch and impaired graft function [1, 4, 6, 7, 14]. Although oversized grafts may anatomically fit within the hyperinflated thoracic cavities of COPD recipients, the recipient’s limited cardiac output may be insufficient to fully perfuse the graft, potentially leading to perfusion mismatch and hypoxia-induced injury [1, 6]. Moreover, the mechanical compression of oversized grafts may hinder microcirculatory flow, exacerbating early graft injury [14].

Conversely, the undersized group exhibited a more favorable PGD profile at later postoperative time points and significantly superior overall survival. One possible explanation is that following pneumonectomy of hyperinflated native lungs, the thoracic cavity undergoes partial remodeling [13, 15]. Moderately undersized grafts may better accommodate this remodeled space without causing compression, thereby facilitating optimal expansion and improving V/Q matching [1, 14]. This may lead to reduced ischemia-reperfusion injury and support better long-term outcomes.

In our cohort, although secondary pulmonary hypertension was more common in the undersized group, the incidence of PGD at 48 and 72 h was significantly lower in this group, which may appear to be paradoxical. This can be explained by the fact that in transplant patients with COPD, replacing an autologous lung severely damaged by emphysema with a structurally normal donor lung may increase the functional pulmonary vascular bed and reduce reperfusion-related injury [16], even when a smaller graft is used. Another reason is that PGD is a multifactorial condition involving ischemia-reperfusion injury, inflammatory responses, and donor-recipient-related factors [17], and size mismatch is only one of these factors.

On the other hand, CLAD is a major determinant of long-term outcomes after lung transplantation and is associated with repeated epithelial injury driven by factors such as acute rejection and infection [18]. These insults promote chronic inflammation, impaired repair, and progressive fibrotic remodeling of the allograft [19]. Although these mechanisms are widely recognised, the onset of CLAD is multifactorial and is influenced by immunological, genetic and environmental factors [20, 21]. Based on this background, while there was no significant difference in the incidence of CLAD between the size-matched groups in this study, multiple factors may have interacted during the development process, potentially contributing to the observed differences in long-term prognosis.

This study has several limitations. First, it was a single-center retrospective analysis, and the sample size—particularly in the undersized group—was relatively small. Due to the fact that only two patients had a pTLC ratio <0.8, we set the cutoff value for undersizing at 0.9. As a result, this study includes very few cases of extreme undersizing, which may have limited our ability to detect its potential physiological consequences. Second, missing data on certain perioperative parameters, such as transfusion volume, precluded comprehensive analysis of all relevant variables. Third, while we attempted to account for baseline differences, unmeasured confounding factors (e.g., renal function, cardiac performance, infection, or malignancy) may have influenced long-term survival. Finally, although some oversized grafts underwent wedge resection, its potential impact on clinical outcomes could not be separately assessed in this study. Graft reduction may be one reason for the poor prognosis of oversized grafts, which is consistent with the previous study [22].

In summary, our data suggest that in bilateral lung transplantation for COPD, modest undersizing may be associated with lower PGD incidence and improved long-term survival, whereas oversized grafts may predispose recipients to early graft dysfunction. Further multicenter studies with larger cohorts are warranted to validate these findings and refine donor-recipient size matching strategies.

In conclusion, undersized grafts could be considered a viable option in COPD patients undergoing bilateral lung transplantation. These results may carry implications for optimizing graft size selection in clinical settings.

## Data Availability

The raw data supporting the conclusions of this article will be made available by the authors, without undue reservation.
